# New insights on affective morbidity and childhood maltreatment

**DOI:** 10.1192/j.eurpsy.2021.121

**Published:** 2021-08-13

**Authors:** V. Kumari

**Affiliations:** Life Sciences, Brunel University London, Uxbridge, United Kingdom

**Keywords:** emotional abuse, Neglect, physical abuse

## Abstract

Childhood maltreatment (physical, sexual or emotional abuse, and physical or emotional neglect, by a parent, caregiver or other adult) is a major problem of global significance. Children who suffered maltreatment of any kind are known to experience a range of mental health problems, including depression, anxiety, psychosis, substance abuse, eating disorders, suicidal symptomatology and personality disorder. These problems often emerge in childhood and last through adulthood to old age. Furthermore, people with a history of childhood maltreatment show a worse-than-usual response to standard treatment approaches to improve their mental health. There is growing evidence that emotional abuse, one of the most prevalent forms of childhood maltreatment, may have the most wide-ranging impact of all maltreatment types on mental health outcomes as it appears to be a transdiagnostic risk factor for several psychiatric disorders, and found to me more strongly associated with development of affective disorders than physical abuse or neglect. There is a need to move beyond simple association studies and shift the research focus on sophisticated multimodal studies to fully understand the psychobiological mechanisms underlying affective morbidity, as well as the protective factors that might promote resilience, in the face of (specific forms of) childhood maltreatment.

**Disclosure:**

No significant relationships.

